# Remarkable Disappearance of a Pessary, and Its Subsequent Removal from the Rectum

**Published:** 1859-09

**Authors:** 


*Remarkable Disappearance of a Pessary, and its Subsequent Removal from the Rectum.* By Dr. LUDERS. (Ed. Med. Journal and Va. Med. Journal.)

A small though elderly lady, who had already borne several children, was seized in autumn 1855 with a complaint in the lower part of the abdomen. The widwife consulted, diagnosed a prolapsus uteri, and proceeded to introduced a pessary. The first tried proved too large, and with some pain and difficulty a less one was introduced. Next morning it was not to be found; and, though nothing was known to come away, she was soothed with the expressed opinion that she must have lost it. The complaint progressed, and assumed all the symptcms of a peritonitis; repeated examinations disclosed a tumor between the rectum and vagina. Half a year after the disappearance of the pessary another physician discovered the tumor mentioned, and a transverse cicatrix in the vagina. For two years she continued to suffer more or less, till in autumn 1857 an opening was formed in the interior wall of the rectum, through which the pessary was at once and easily removed. It was a common caoutchouc ball, stuffed with hair, 3½ inches long, 2½ broad, and 1 inch thick, and with a small opening in the middle. The patient made a rapid recovery, and it is remarkable that no tract of prolapsus was ever discovered. (The foregoing, says the Rep., affords a melancholy instance of the painful sufferings to which even educated females are exposed, from the prevalent habit of consulting *midwives* in sexual complaints, and how necessary it is to forbid such to introduce pessaries under pain of severe punishment.) *Deutshe Klinik—Prager Viljschft.*

We don't know what our esteemed friend, the author of "Horæ Subsecivæ" will say to this. In no country of the world are midwives so carefully educated as in Germany; yet the foregoing case distinctly shows how incompetent they are to make the simplest diagnosis, or perform the easiest operation, and the remarks appended prove that such ignorance is no rarity. And, as additional proof, and from an unprejudiced source, we may add that the late lamented Kolletschka, professor of medical jurisprodence at Vienna, was in the habit of saying in his lectures, that broken arms, and legs, and heads left behind in the uterus, were not at all unfrequent, but often happened during the first years, particularly, of an Austrian widwife's practice. Yet no-where do midwives receive a better education, or have a larger practical field for instruction, than in Vienna.

